# “Sometimers, Alzheimer’s? I love that! That’s definitely me”: Readers’ Responses to Fictional Dementia Narratives

**DOI:** 10.1093/geront/gnad055

**Published:** 2023-05-12

**Authors:** Gemma M Carney, Jane Lugea, Carolina Fernandez-Quintanilla, Paula Devine

**Affiliations:** ARK Ageing Programme, School of Social Sciences, Education and Social Work, Queen’s University Belfast, Belfast, UK; School of Arts, English and Languages, Queen’s University Belfast, Belfast, UK; Department of English and German Philology, University of Granada, Campus Universitario de Cartuja, Granada, Spain; ARK Ageing Programme, School of Social Sciences, Education and Social Work, Queen’s University Belfast, Belfast, UK

**Keywords:** Age studies, Cultural gerontology, Literature, Metaphor, Philology

## Abstract

This article presents findings from an interdisciplinary project which invited readers to experience the impact of dementia via fictional characters’ narratives. Combining methods from critical gerontology and literary linguistics—a field that examines the language of literature—we undertook an empirical reader response study of dementia fiction. We constructed a large corpus of dementia fiction; selecting 12 extracts, each containing first-hand, focalized accounts of fictional characters’ experiences of living with dementia. Readers (31) were purposively sampled for 4 separate reading groups—student social workers (9); general public (9); family carers (6); and people with dementia (7). Over 6 weeks they engaged in separate, facilitated, on-line group discussions of extracts. Discussions were independently coded using *ATLAS.ti*.

Although readers from all 4 groups reported that fictional characters drew them into the internal life of someone with dementia, some carers questioned whether fictional characters’ experiences were plausible. Readers with dementia recognized themselves in the extracts; viewing fictional characters as eloquent envoys of their lived experiences of diagnosis, social isolation, loss of language, and use of humor.

Fictional characters offer an entry point for understanding contrasts in caregiver and care-receiver experiences of dementia. Fictional characters are potentially useful for moving dementia narratives beyond monstrous cultural metaphors and onto a disability-based rights agenda.

Working across disciplines, humanities scholars have enriched the language and conceptual schema around dementia narratives (see Bitenc, 2012, 2020; Swinnen, 2013; Swinnen & de Medeiros, 2018; Twigg & Martin, 2014). Fields of exchange across arts, humanities, and science offer means for understanding and amplifying the perspective of people with dementia (Basting, 2020; Basting et al., 2016). Arts and dementia scholar Zeilig has worked with disabilities scholar Shakespeare in calling for dementia to be re-assessed as a disability (Shakespeare et al., 2019). The disability movement’s mantra “nothing about us without us” has appeared in the context of research undertaken by the Dementia Enquirers (dementiaenquirers.org.uk), a group of people with dementia who publish research about their experiences of living with dementia as authors rather than subjects of study (Davies et al., 2021). Their work builds on that of Bartlett (2014, 2022) who documents the efforts of activists with dementia to achieve citizenship rights; and Basting (2009, 2020) whose pioneering dementia research re-imagines care relationships.

Meanwhile, the search for the “person in dementia” (Kitwood, 1997) has been extended into cultural and media studies. Clarke (2006, p. 269) revealed that the person with dementia was entirely absent from mass print magazine coverage of Alzheimer’s disease over a 10-year period (Clarke, 2006, p. 269). Harvey and Brookes’ (2019) study of internet stock imagery of dementia found “older people with dementia to be represented in objectifying and de-humanizing terms, emphasizing disease and deficit at the expense of the whole person…” (Harvey & Brookes, 2019, p. 987). Bailey et al. (2021) used a combination of corpus linguistics and critical discourse analysis to examine representations of dementia in the British press, concluding “the most salient discourse…was the portrayal of dementia in biomedical terms” (Bailey et al., 2021, p. 362).

This cultural status of dementia as “a complex, unknowable world of doom, aging, and a fate worse than death” (Zeilig, 2013, p. 262) provides the backdrop for our project, *Dementia in the Minds of Characters and Readers*, in which we employ fictional literary language to study an illness that degrades language and cognitive power. The project builds on Bitenc’s (2020, p. 7) contention that “the novel provides an inroad into understanding the life world of others,” though we acknowledge that identifying with fictional characters is not straightforward (Eder et al., 2010).

We focus on dementia fiction because it has seen something of a boom in recent decades. Bestsellers such as Emma Healey’s *Elizabeth is Missing* and Lisa Genova’s *Still Alice* have been dramatized in film. As the experts on our team are literary linguists rather than film specialists we focused on the language of fictional characters in prose rather than film form. *Still Alice* is an example of creative exchange between humanities and science. Genova’s neuroscience career led her to conclude that a fictional character could help her to better communicate living with dementia. *Still Alice* was on the *New York Times* bestsellers list for over 40 weeks. Once these fictional accounts reach a mass audience they are influential in informing public understanding of dementia (Wade, 2015).

Our project explored how fictional narratives portray the experience of characters with dementia. How those narratives are read and interpreted by real readers is indicative of public “dementia narratives”; that is, “the stories we tell each other about dementia” (Bitenc, 2020, p. 9). This article examines readers’ responses to extracts from fiction, aiming to uncover how literary representations may correspond with real-life understandings of dementia.

From the perspective of literary theory, the notion of “character” is not unproblematic as characters are “highly complex objects”: for example, they remind us of real people and seem to exist in reality, but we do not meet them in the street and cannot interact with them directly (Eder et al., 2010, p. 3). Despite their complexity, we take the concept of “character” to fulfil criteria such as “being animate, having an intentional mind, being able to act, being humanlike and having person status” (p. 10). All of the characters with dementia we included in this project offer some enlightening or engaging insight into what it might be like to *be* that person with dementia. Arguably, this reliance on fiction is a limitation of the project, but we believe that it is also an advantage. It is through creative language that subjective experience can be brought to life for readers. Fictional characters are well-rounded. All the characters we include have flaws, personal histories, or relationships which are at times rewarding and at others draining. These characters are more than their dementia diagnosis (see Table 1). Next we describe the methods used to select texts, fictional characters, and readers.

**Table 1:** Texts Selected

Author and publication year	Title of book	Character with dementia	Background given to reading group
Rill, Eric (2015)	*An Absent Mind*	Saul Reimer	Saul Reimer is 71, Jewish-Canadian, and is married to Monique. He was recently diagnosed with dementia.
Healey, Emma (2015)	*Elizabeth is Missing*	Maud Palmer/Horsham	Maud has come to a department store with her daughter, Helen, and granddaughter, Katy, but now she is lost. She is often plagued with memories of her sister Sukey, who disappeared when she was young and is still missing.
Dean, Debra (2006)	*The Madonnas of Leningrad*	Marina Buriakov	Marina was an art museum guide in Leningrad, Russia, and was sieged there during WW2. Now she is 82 and lives with her husband Dmitri in the USA, where they raised their children.
Harvey, Samantha (2010)	*The Wilderness*	Jake Jameson	Jake is looking at old photos. He was married to Helen, but he often remembers Joy, who he had a brief affair with early in his marriage. Joy was the daughter of his step-father, Rook.
Genova, Lisa (2007)	*Still Alice*	Alice Howland	Alice is in the later stages of early-onset Alzheimer’s Disease. She is with her daughters: Anna, who has just had a baby and Lydia, an actress.
LaPlante, Alice (2012)	*Turn of Mind*	Jennifer White	Dr. Jennifer White is a retired surgeon, now living with dementia in a care home.
Bernlef, Josef (pseudonym of Hendrik Jan Marsman) [trans. by Adrienne Dixon] (1988)	*Out of Mind*	Maarten Klein	Maarten and Vera raised their children in the USA, but they are Dutch. Maarten is retired and has not been feeling himself recently.
Hepworth, Sally (2017)	*The Things we Keep*	Anna Forster	Anna has early onset Alzheimer’s Disease and lives in a care home. Today her twin brother, Jack, and favorite nephew, Ethan, are visiting.
Smith, Ali (2012)	*There But For The*	May Young	May is aged 84 and has dementia. She is in hospital because of a UTI and delirium. She has been given medication, but has hidden the pills in her hand.

## Methodology

### Corpus of Dementia Fiction

First, the second author, a specialist in cognitive stylistics and literary language, constructed a large corpus of 400 000 words of dementia fiction. For a detailed exposition of the methodology for this first phase of the project see Lugea (2022). From this corpus, she undertook a systematic process of selecting relevant texts according to the following criteria: 1. Contemporary (i.e., published in last 35 years); 2. Fictional (not autobiographical); 3. Prose; 4. In the English language; 5. Featuring a main character with any type of dementia; 6. Access to the internal perspective of the character with dementia either through first person account (with the character with dementia as narrator), or through third person account (with a narrator reporting on characters and events) via Free Indirect Style or psycho-narration. The latter are two ways for narrators to present the contents of characters’ minds, something which is unique to fiction as in real life we cannot access other people’s minds in the same way (Palmer, 2004). In FIS the narrator’s report blends with the character’s consciousness. In contrast, in psycho-narration, a narrator can verbalize mental activity of which the character may not be conscious. We were also interested in linguistic features used by authors to depict dementia (e.g., underlexicalization—the character’s inability to recall a specific word, using a marked alternative word or phrase). We decided to focus on fictional characters rather than autobiographical accounts because we wanted to be able to explore how “*fictional* language represents the cognitive experience of dementia” (Lugea, 2022, p. 169, emphasis added).

From this corpus, author 2 selected 12 novels that met all 6 criteria. The whole team then worked together to select extracts from those novels, each containing first-hand, focalized accounts of fictional characters’ experiences of living with dementia. This shortlist was reviewed and 12 extracts were chosen—2 extracts for each of the 6 reading group meetings held (Table 1). Extracts were chosen if they included metaphors or other linguistic devices which allowed the reader to access the character’s experience of living with dementia, referred to as their “dementia mind style” (Lugea, 2022, p. 175).

Despite best efforts to achieve an ethnically diverse range of fictional characters with dementia to include in our study, most were female, old, white, or middle-class. A common form of racial diversity in the novels we examined included paid carers from black/ethnic minority groups. In part, this is a result of the approach taken in compiling the corpus of dementia fiction. By limiting the texts to those published in English our study is not generalizable beyond “Anglophone cultural representations of dementia” (Lugea, 2022, p. 173). Although this is a limitation of the project, it reflects the genre more generally and so we have sought to address this via the publication of an anthology of dementia fiction which commissioned fourteen dementia stories including one by a Syrian-Irish author and another by a British-Ghanaian author (see Carson and Lugea, 2022, p. 5).

The criteria for extract selection were different for the reading group containing people with dementia. Dementia Engagement and Empowerment Project (DEEP, 2013) guidance provided by Dementia Northern Ireland (NI), who worked with us throughout the project, recommended we include slightly shorter extracts for readers with dementia, as they can experience lapses in concentration. So, readers with dementia dealt with one extract in each reading group session. Other reading groups all read exactly the same two extracts per session. Support was provided to them by Dementia NI. Ethical approval was secured from the university ethics committee.

### The Reading Groups

More detail on methods, including socioeconomic status (SES) and educational qualifications of readers are available in the Supplementary Material. Here we offer a succinct summary.

We wanted to compare how people with different experiences of dementia were affected by reading the extracts. Readers (31) were purposively sampled for four separate reading groups—student social workers (9); general public (9); family carers (6); people with dementia (7). Students were first-year undergraduates and were recruited via the program director for social work at the university. Other participants were recruited via Dementia NI and Alzheimer’s Society, and existing networks of outreach officer, Jan Carson. Carers and people with dementia were recruited because of their lived experience of dementia, but some participants in student and general public groups transpired to have family experience of dementia. All but one participant was white and English speaking, a product of the homogeneity of the population of NI, which is 96.5% white according to 2021 census (Northern Ireland Statistics and Research Agency, 2022). Recruiting people with dementia and carers to take part in six weekly reading groups during a pandemic was challenging. However, every effort was made to have as diverse a sample as possible in terms of educational qualifications and SES. Table 2 demonstrates that both groups were diverse in terms of SES and there were two women for every male participant.

**Table 2.** Readers’ Demographic Characteristics

Group	Gender	Age	Attendance	Pseudonyms	Socioeconomic status	Educational level
A: Student social workers (9)	6 female, 3 male	7 (18–29), 2 (39–49)	7 attended at least 4 session	Sarah-Jane, Jamie-Lee, Kylie, Maura, Ciara, Ashling, Brian, Kieran, Timothy	All student social workers.	All in higher education, all had A level, 2 had undergraduate degree; 1 had postgraduate degree.
B: General public (9)	8 female, 1 male	18–69	8 attended at least 5 sessions.	Poppy, Nora, Marianne, Annie, Julia, Lorraine, Ann, Nicola, Mike	Psycho-therapist, civil servant, shop owner, publishing, retail, teacher.	All had A level or higher: 4 with undergraduate degree 3 with postgraduate.
C: Carers (6)	4 female, 2 male	40–79	6 attended at least 5 sessions.	Mary, Jemima, Monica, Amy, Matt, Philip	Hospitality, Engineering, Self-employed, human resources, nursing.	2 with GCSE 3 with A level 1 with undergraduate degree.
D: People with dementia (7)	4 female, 3 male	50–79	4 attended at least 5 sessions.	Andy, Naomi, Danny, Will, Joanna, Anthony, Sheila.	Building, Hairdressing, Driving, Disability support worker.	3 had no qualification,1 had GCSE; 2 had undergraduate degree.

*Notes*: Education qualifications in the UK have nine levels. General Certificate of Secondary Education (GCSE) are levels 1–2; A-level graduates are level 3 or 4. Undergraduate university degree is level 6, postgraduate degree is level 7, and doctorate is level 8. See Supplementary Material for full explanation.

Over 6 weeks the readers engaged in separate, facilitated group discussions of extracts. Due to COVID-19 restrictions, weekly reading groups were held via meeting platform, Zoom, March—May, 2020. The format and materials for all sessions were identical, to ensure consistency (Fernandez-Quintanilla, 2020). Each session began with a recording of the extract being read aloud. Readers were asked to mark up the hard copy they had been sent by post. Then, before any discussion took place, readers were asked to fill in an on-line questionnaire exploring their reactions and feelings about the characters. Next, we invited everyone back into the virtual room to discuss the extracts.

Each reading group was facilitated by two researchers. We employed a “naturalistic” approach to reader response (Hall, 2008), and so tried not to influence the discussion. Rather, we asked open questions like “to what extent could you understand Maarten’s perspective?” or “How did Saul’s story make you feel?” The discussions were professionally transcribed and anonymized. Carney and Lugea undertook a year-long independent coding regime. Full details are in the Supplementary Material.

## Results

All four groups concluded that reading about the internal life of fictional characters with dementia allowed them to learn about dementia and to ask difficult questions of themselves and others. Stylistic devices in the extracts such as metaphor, under-lexicalization, dramatic irony, humor, time slips, and narrative strategies, were effective in allowing the reader to enter the “life world” of someone with dementia (Bitenc, 2020, p. 7). The extent to which readers responded to and bought into the fictional narratives was related to their previous experience with dementia. Given the dearth of attention paid to the perspective of people with dementia in any research on the topic, we dedicate most space to reporting the views of readers with dementia in this article (Davies et al., 2021). Before sharing a detailed textual analysis, we present the main findings from independent coding of the reading group discussions for all four groups (Table 3).

**Table 3.** Overview of Top Scoring Codes by Reading Group

	Name of code and groundedness score (i.e., number of quotations coded to that code)					
	Attributing feeling to character with dementia (Gr = 428)	Response emotional (Gr = 297)	Participant agreement (Gr = 222)	Reader compares fictional character with dementia with person in real life (Gr = 219)	Positive evaluation of text (Gr = 207)	Response relate (Gr = 212)
Student social workers (A)	182	61	49	36	20	30
General public (B)	134	130	62	36	68	41
Carers (C)	91	106	94	155	80	81
People with dementia (D)	40	8	73	3	48	78
Totals	447	305	278	230	216	230

*Note:* Group D, people with dementia read only six of the 12 extracts so report smaller groundedness scores.

Using *ATLAS.ti,* this table was compiled by producing a code co-occurrence table for each of the reading groups, selecting the most populous codes, according to groundedness score (Gr.). The groundedness score is the number of quotations that coders have linked to a certain code (i.e., the frequency count of each code). Codes with larger Gr. scores have more quotations attributed to them. They are therefore the codes on which coders had the most agreement, though bear in mind that these are affected by the number of participants in each group and the fact that readers with dementia read 6 rather than 12 extracts. So, the first column of Table 2 shows that across all four groups readers attributed feeling to the character with dementia. This is expected as the extracts they read were completely focused on the internal narration of a character with dementia. The score of 428 shows that coders found 428 quotations where readers attributed feelings to the fictional character with dementia; that is, readers grasp how characters feel and ascribe their emotional states. For example, “Like, this old lady she is standing there, you know, panicked, crying, she’s clearly lost and confused” (Kylie on Maud, *Elizabeth is Missing*). We take this to mean that the readers found the characters plausible and believable. Column two shows that coders found readers reported having an emotional response to the extract. This code referred to the explicit reaction of readers when they said something like “I was just really disappointed at the treatment she received” (Aisling on Maud, *Elizabeth is Missing*). Column three shows that there was a high level of agreement between participants.

The coders found that readers often compared the fictional character with dementia to someone in real life. This may be related to high numbers of participants who had at least some family experience with dementia (column 4). The readers with dementia did not score highly in this category because there was another code “compare self with character with dementia” (Gr. 97) which they aligned with. The final two columns demonstrate that readers remarked on how the text was true to life, for example, “I thought it was very lifelike…to the experience I’ve had of caring for someone with dementia” (Philip on Saul, *An Absent Mind*). Finally, readers often stated how they could relate to the text. Next, we present our analysis of the reading group discussions.

### Analysis of Reading Groups

#### Readers with dementia

We set out to treat people with dementia as equal participants in this study and so have purposely not focused on elaborate accounts of their illness. However, they had been diagnosed with at least one form of dementia before taking part in the study. One person was recently diagnosed but the other six participants had been diagnosed between 3 and 9 years previous. Table 4 offers some examples of codes and quotations from our codebook.

**Table 4.** Readers With Dementia—Codes and Examples From Codebook

Code	Code definition	Example of quotation from Codebook, Reader and Character.
Attributing feeling to character with dementia	Reader attributes feelings to the character with dementia, either explicit in the extract, or imagined.	“Anna seems very angry and frustrated both with, and by, her condition. She swears in frustration at not being able to recall names and places” (Andy on Anna, D64, p. 169).
Comparing Self with character with dementia	Reader compares themselves to character with dementia.	“I could relate to that, definitely. I could see myself sitting there in the same places. Saul Reimer, it’s definitely accurate” (Andy on Saul Reimer, D8, pp. 149–164).
Evaluation of text—positive	Reader gives a positive evaluation of extract.	“That was a very good extract” (Danny on Marina, D41, p. 19).
Style—under-lexicalization	Reader refers to loss of specific/accurate language as symptom of dementia.	‘She wanted she wanted to find a “whirly-thing” (Naomi on Anna, D64, pp. 120–121).
Insight into character/person with dementia’s perspective	Focused or elaborate account of the person with dementia’s experience—character and/or real life.	“And then, and then the frustration and the confusion that the noises are causing. And then the pleasure sort of, of being asked to sit and listen, and to give an opinion of it” (Naomi on Marina, D41, pp. 195–196).

*Notes*: D64: 169 is the exact reference to the quotation in our *ATLAS*.*ti* data set which are fully explained in the Online Supplementary Material.

Before beginning to code the transcripts of the reading group discussions, Carney spent many hours listening to recordings of the discussions. The participants in our study who have a dementia diagnosis were articulate, easy-going, and witty. They used black humor when sharing experiences of living with dementia. They were not easily upset by reading about dementia, with groundedness scores for codes like “feeling pessimistic about future” (Gr. 13) and “response emotional” (Gr. 8) being much lower than more neutral categories such as “response relate” (Gr. 78). The readers with dementia also tended to score highly on “participant agreement” (Gr. 73), often supporting one another when one of them struggled to make a point. Sometimes this mutual support extended to the character with dementia, coded as “comparing self with character with dementia” (Gr. 48). During session 2 Naomi advocates for Alice, the main character in *Still Alice*. In the extract, Alice is holding her baby grand-daughter, whose mother administers some stern advice that Alice is not to let the baby eat her necklace. As well as putting herself in Alice’s shoes and interpreting Alice’s behavior for the group, Naomi points out that sometimes it is not quite clear whether it is age or dementia diagnosis that prompts younger people to patronize.

…a lot of it rang sort of very true to me, you know about, ‘don’t let the baby suck that’… I don’t know… whether it is the fact that, because of your dementia they sort of think, ‘I have to remind her here don’t allow the baby to do this or don’t-,’ or whether it is just the way they would treat anybody, because they know how to bring the baby up better. (Naomi on Alice, D41, p. 146)

Later in this extract, Alice’s younger daughter, Lydia, with whom she had a fractious relationship, asks Alice to listen to her deliver a monologue from her acting class. Alice cannot remember her own daughter’s name, so she refers to Lydia as “the actress.” After the brief performance the actress asks “Okay, what do you feel?” to which Alice replies “I feel the love. It’s about love.” (Genova, *Still* Alice).

Alice’s capacity to sense a mood even when she could not understand the words used to describe it is verified by Joanne, the participant with most advanced dementia who when asked how that extract made her feel says “I could feel the love between them” (Joanne on Alice, D41; 202). Danny then agrees that the treatment of Alice by her two daughters—one who patronizes her and another who asks her opinion—is fairly typical. The session concludes with Naomi expressing her astonishment that now, in week four, opinion between the group is undivided. “These sessions just really fascinate me how on every occasion, everybody’s been on exactly the same page” (Naomi, D41, p. 238). The group later goes on to agree that people without experience of dementia might struggle to understand the extract. However, they did argue that reading a whole book, not just an extract, like *Still Alice*, might benefit their carers. Danny remarks, “to read something like that there and especially read a full book of that there, it would give them a better insight into our conditions, I think” (Danny, D41, p. 238).

The need for carers to get a better insight into living with dementia was taken further the following week through a spontaneous discussion of the story of Maarten from *Out of Mind* as a means of explaining what it feels like to live with dementia to others (coded as “insight into living with dementia,” Gr. 54). Readers explain how their Dementia NI Empowerment group is important as it allows them to spend time together, i.e., with people who understand how they feel. “It took some time but then once I joined Alzheimer’s Society group and then, hence went out to join the beginnings of the group that set up Dementia NI, you know, that my, you know, my life was what would you say really? I don’t know what the word is that I would need to be describing it as, but it really was, made an awful difference to me” (Sheila, D8, pp. 173–174).

Throughout the six sessions, we observed that some of the readers with dementia tended to find one character with whom they could identify. Andy identified with Maarten from *Out of Mind,* saying; “I can relate to Maarten so comfortably. I feel comfortable as if I’m sitting talking to the man. Very happy in his world. I’m happy.” Andy draws out the book’s use of metaphors of the sea. He observes that Maarten “compares himself to a ship at the sea, where he seems to have lost his meaning. And then all of a sudden it feels as though he’s been given a purpose again” (Andy on Maarten, D52, p. 42).

A number of times readers with dementia drew our attention to the fictional characters’ capacity to shift in time, from the present into the past and back again. Danny takes up the case of Marina in *The Madonnas of Leningrad.* In the extract, Marina floats back to a time she spent salvaging paintings during the Seige of Leningrad in 1942. She is then suddenly back in her contemporary kitchen, and cannot remember whether she has had breakfast or not. She has forgotten that she and Dimitri are going to a family wedding that weekend. When she struggles to remember the names of close relatives, Dimitri despairs and Marina’s internal narration is that she can “see the ghost of despair in his eyes.” This phrase rings true for Danny who shares an anecdote of having a similar memory lapse only the night before. He is keen that others understand the effort Marina will be making to remember: “She is trying so hard in her own mind to remember things…she is looking into his eyes and she can see the despair in his eyes. And it’s a horrible feeling to be like that” (Danny on Marina, D19, p. 97). Marina’s case is further advanced by Naomi who explains the importance of Dimitri’s gentle use of reminders like colors and patterns; “whenever she was given gentle, sort of reminders, or hints to help her remember, like the dress she was wearing… and that all of a sudden brought that memory back, and she could recall in detail, the dress, and that in turn stimulated another memory …” (Naomi on Marina, D19, p. 89).

As the weeks passed, Naomi, Andy, Danny, and Anthony discussed how the research was making them realize they missed reading. Andy explains how he has adapted by using audiobooks: “I just order a book and I sit and listen to the whole book one evening—if you ask me tomorrow what it’s about, I probably haven’t got a clue like, but I enjoy listening” (Andy, D8, p. 283).

Readers with dementia also identified strongly with Saul Reimer, *An Absent Mind,* his caring family, and his need to use yellow notepads as an *aide memoir*: “there was some things I can relate to—see the bit about the big yellow pad … Sometimer’s Alzheimer’s? I love that! That’s definitely me” (Will on Saul, D8, p. 85). Saul Reimer appeared as a particularly plausible character. Partly because he was witty, or perhaps because the extract outlined the process of sharing a dementia diagnosis: “See it’s very, very hard after diagnosis, because your world has just stopped you know, acceptance is the most important part of the whole journey” (Andy on Saul, D8, p. 123). Andy explains how he copes with the reality of dementia, which becomes a fact of life: “I don’t worry about dementia. I just get on with my life and whatever is to be is to be!” (Andy, Saul, D8, p. 123). This view is shared by other readers with dementia and is repeated regularly.

#### Readers who are carers

Table 5 outlines some examples of carers’ responses to the extracts.

**Table 5.** Readers Who Are Carers—Codes and Examples From Codebook

Code	Code definition	Example of quotation from codebook, reader, and character
Compare with real person—character with dementia	Reader compares fictional character with someone from real life.	“Well, I I have to say I found the turn of phrase quite humorous at times, so I had quite a few sorts of wry laughs erm… my mother wasn’t a doctor, but she reminded me very, very much of my mother, her spirit and her determination” (Amy on Dr. White, D44, p. 50).
Lived experience—family/carer perspective.	Reader offers example or insight into dementia from a carer’s perspective.	**“**And that whole business of the look on your face, there were times when I would capture, she would look at me and I would try hard, so hard not to have an expression on my face that might upset her, or my reaction, but sometimes you just can’t help it” (Monica on Marina, D18, p. 28).
Comparing with self—caring character	Reader compares caring character with themselves.	“So all of this I could really relate to keep going back to my own situation” (Monica on Jennifer, D44, p. 97).
Response—questions fictional account	Reader openly questions the validity of fictional account of living with dementia compared with real life experience.	**“**I just don’t think that those those words would be available to somebody. Certainly, you know, with Alzheimer’s dementia, I’m not sure with other dementias, but you know, that is poetic license” (Mary on Jake, D29, p. 129).
Response—emotional	Reader reports emotion triggered by extract.	“So that one really kind of sparked a lot of frustration and anger in me, because I still think, which was very funny at the time, because in the bank, they had the dementia friendly badges for sale” (Monica on Maud, D11, pp. 70–71).

*Note*: D64: 169 is the exact reference to the quotation in our *ATLAS*.*ti* data set which are fully explained in the Supplementary Material.

Readers who are carers were more likely to respond emotionally to the extracts than other groups. They shared the most pessimistic interpretations of the actions of our fictional characters. In the fourth session, we included a challenging extract from *Turn of Mind*, where the protagonist, Dr. Jennifer White, a retired neurosurgeon who has dementia, describes in detail the alienation she feels at the hands of anonymous carers in a residential home:

Not milk. *Coffee*, I say, but no one is listening. That’s the way it is here. People will say anything, promise anything. You can ignore the words, even on the days when you can retain them, because you need to keep your eyes on their bodies. Their hands most of all. The hands don’t lie. You watch what they are holding. What they are reaching for. If you cannot see the hands, that is the time to be concerned. The time to begin screaming (LaPlante, *Turn of Mind*, extract 4b).

The carers had a strong reaction to this extract. “I struggled with it because it was so hard hitting, I think really, you know … how on earth am I going to make this eventually, you know, a good experience for Peter?” (Mary on Dr. White, D44, p. 62). Mary is upset by the extract but also questions it, appealing to the other carers in the group whose loved one is already in a care home: “I am sure you all got some very nice carers for your, for your loved ones. And it but it just scared me a little bit to be honest …” (Mary on Dr. White, D44, p. 62).

Throughout the study, carers were the group that questioned the internal monologue of the characters with dementia most often. Typically they questioned the capacity of the fictional character to operate machinery or perform a task. In Session 2, they read an extract where Jake, *The Wilderness*, is completing a timeline of his life on the advice of his doctor. The carers suggest that it is unlikely that Jake could complete this task without help. Philip says: “Feed it to the dog probably!” (Philip on Jake, D33, p. 181).

In Session 2, the group read an extract from *Madonnas of Leningrad* where Marina is asked standard questions at a memory clinic. She and her husband-carer, Dimitri are despatched, without help, given only advice to “be patient and vigilant.” Matt says: “I could actually feel myself getting angry with this because I’ve lived this… A sheet of prescriptions, and patience and vigilance. It does not come anywhere near what it should have been… Because after all that upset it may have caused, that’s up to the carer then to go and try and calm things down and make everything relaxed again” (Matt on Marina, D22, pp. 93–94).

However, like the readers with dementia, the carers identified strongly with Saul Reimer, the originator of the “Sometimers, Alzheimer’s” phrase in *An Absent Mind*, even picking out the same quotation: “That brilliant quote at the end of ‘the some-timers not Alzheimer’s’ …that is exactly how my wife reacted. Well, just because I forget some things doesn’t mean I forget everything …” (Philip on Saul, D7, p. 117). However, Monica and Matt then go on to highlight the suspicion and fear that Saul must feel, signified by the title of the extract: “The Lynch Party”—“So immediately just with that title, raised lots of emotion for me of how it was right in the beginning, trying to get her to realize there was a problem” (Monica on Saul, D7, p. 143). Monica goes on to explain how difficult the situation is for “us” trying to get her mother to realize there was a problem. Likewise, Jemima sympathizes and “feels for Saul,” but concludes that “reading a piece like that, oh, my God, you’re right back in the minute …” (Jemima on Saul, D7, p. 155).

The carers read the extract from *Still Alice* described in our analysis of readers with dementia. They identify Lydia as Alice’s carer, despite the fact that she is only referred to as “the actress” in the extract. They focus in on Alice’s loss of language as a valid example of what typically happens as the disease progresses. “I agree with the loss of language and describing functions for items … yesterday, I was grating some cheese. And he asked for the scraper thing, you know?” (Mary on Alice, D40, p. 103). The overall impression from our coding is that carers are immersed in their own experience, and struggle to see beyond that, particularly when they are still in the act of caring for a loved one with dementia.

## Discussion

The aim of the study was to compare different readers’ responses to fictional characters’ experiences of living with dementia. The objective is not to set the groups up in conflict, rather we focused on the contrast between carers and readers with dementia because it was an overwhelming finding of our analysis. Also, we believe that these findings have important implications for dementia researchers’ efforts to challenge “tightly told tragedies of dementia with science as hero” (Basting, 2009, p. 35). Like other disabled people facing down the medical model of impairment, part of the challenge for people with dementia is to find a way of being heard by those who love them most. Having an opportunity to voice their views without worrying about hurting the feelings of carers is valuable. People with dementia reported this, spontaneously and repeatedly, during our sessions. It is also worth noting that the carers and people with dementia were broadly similar in terms of educational qualifications and SES. A much larger project would be needed if we were to offer conclusive evidence of how these factors may have influenced their capacity for literary analysis.

In this article, we found that carers used the extracts as a foil against which to reason. Carers were more skeptical of the experiences of the fictional characters discussed, but this did not diminish their agreement that many of the scenarios, language, and behavior in the fictional extracts reflected back their experiences as carers. Those whose relative was in the earlier stages of the illness found some of the extracts alarming or upsetting as they laid out a negative view of the future. The carers empathize most readily with the caring characters, often questioning the views or actions of the character with dementia, even whilst endorsing the overall text. Their perspective on dementia was deeply personal. Their lived experience of dementia was as a carer who was often isolated, lacked resources, and felt left behind when their partner or relative became progressively lost to dementia (Basting, 2020).

Conversely, the readers with dementia advocated for the fictional characters in the extracts, often filling in blanks or explaining behaviors. This gap may, in some parts, be explained by the fact that some extracts read by the carers group were not read by those with dementia. However, in the final analysis, we find that this difference is more fundamental, particularly given contrasting responses to the same extract (such as the example of Alice and “the actress”). At its core, the “stories we tell ourselves about dementia” (Bitenc, 2020, p. 7) differ depending on our lived experience. Our findings concur with Basting (2009, p. 78) who concluded that some carers who might be in the “grip of grief … hold tightly in their minds to the person as he or she once was” (Basting, 2020, p. 78). For carers, who face the disease as something that is, on a daily basis, diminishing the capacity of a fully productive, adult loved one, dementia more closely resembles the monstrous metaphor identified by Zeilig (2013) and disseminated widely across popular culture and public life.

On the other hand, the internal perspective on dementia has a different quality. The assumption that people with dementia cannot think or feel is based on their perceived loss of language (Bitenc, 2020). The eloquent and enthusiastic espousal of the fictional narratives by people with dementia in this study calls this into question. Once provided with language, scenarios, and an opportunity to discuss their experiences without carers present, readers with dementia provided a vivid, humorous, and articulate account of their inner lives. As the weeks progressed they became more confident with the method, citing the text and identifying metaphors and other ruses used by authors to depict dementia. They were able to take a number of different perspectives, depending on their individual experiences and their own personality. They explained how the responses of carers and families to their illness were challenging from diagnosis onwards. They expressed a high level of agreement amongst themselves. They mentioned how pleased they were that we had sought their opinions. In short, their engagement with the characters, their use of Zoom, and their analysis of the extracts surpassed all expectations of a group of readers already diagnosed with a cognitive impairment. This echoes Rimkeit and Claridge (2017), p. 18) who found that “there was fluent discussion, with play on words and curiosity about the use of language” in readers with dementia. Taken with Basting’s (2020, p. 49) contention that creativity is about working around problems collaboratively, the evidence suggests that shared reading groups between people with dementia and their carers could unleash the potential of reading fiction together as a form of mutual care.

In summary, our extensive coding of different groups of readers encountering and re-encountering dementia in different fictional guises, using a range of narrative styles and linguistic tropes has thrown up a contrast between participants who have direct experience of dementia and those whose knowledge is fleeting or distant. Family carers have deep knowledge of what it is like to love someone with dementia. Their knowledge is personalized and specific to their loved one, their own personality, and the relationship they shared before diagnosis. The carer’s perspective is completely valid, but qualitatively different from that of the cared for.

## Conclusion

Fictional characters allowed readers to openly and frankly discuss what it might be like to live inside the mind of someone with dementia. The evidence provided by our readers with dementia shows that (a) they want to be asked their opinion; (b) they have found innovative ways of coping with their diagnosis; and (c) they want carers to try to *understand* as well as care for them. Lastly, they want to read. The readers with dementia enjoyed using audio recordings to help them articulate feelings and experiences. When this evidence is laid out alongside reports from the Dementia Enquirers (Davies et al., 2021) and Bartlett’s (2014), it appears that even years after diagnosis, people with dementia can articulate their experiences. The onus is on society to provide creative means for them to do so (Basting, 2020). In this, our research follows Basting’s (2016) theatre-based work in care homes which demonstrated how humanities can help us communicate with someone who no longer has memory. Our findings also build on Swinnen’s work and on Zeilig’s efforts to question cultural metaphors which pay more attention to the disease than the person. Once we are willing to listen to first-person accounts of living with dementia, we “look beyond the cultural stereotype” (Swinnen, 2013, p. 121) and thus come closer to finding the “person in dementia” (Kitwood, 1997). People with dementia begin to resemble other disabled advocates and, the argument that they deserve the same rights as any other disabled people, gains ground.

## Supplementary Material

gnad055_suppl_Supplementary_Material

## Data Availability

The research for this study has been registered with the Queen’s University Belfast’s (QUB) Human Subjects database. It has also been through a rigorous system of ethical approval under the university’s research governance policy (see https://www.qub.ac.uk/Research/Governance-ethics-and-integrity/Policies-procedures-and-guidelines/). At this time the data are not available to other researchers for replication purposes as the original research team has not yet finished their own analysis. However, we do plan to share our codebook in a form that protects the anonymity of participants in the future.

## References

[CIT0001] Bailey, A., Dening, T., & Harvey, K. (2021). Battles and breakthroughs: Representations of dementia in the British press. Ageing & Society, 41, 362–376. doi:10.1017/s0144686x19001120

[CIT0002] Bartlett, R. (2014). Citizenship in action: The lived experiences of citizens with dementia who campaign for social change. Disability & Society, 29(8), 1291–1304. doi:10.1080/09687599.2014.924905

[CIT0003] Bartlett, R. (2022). Inclusive (social) citizenship and persons with dementia. Disability & Society, 37(7), 1129–1145. doi:10.1080/09687599.2021.1877115

[CIT0004] Basting, A. (2009) Forget memory: Creating better lives for people with dementia. John Hopkins.

[CIT0005] Basting, A. (2020) Creative care: A revolutionary approach to dementia and eldercare. Harper Collins.

[CIT0006] Basting, A., Towey, M. & Rose, E. (2016) The Penelope project: An arts-based odyssey to change elder care. University of Iowa Press.

[CIT0007] Bernlef, J. (1988) Out of mind. D. R. Rodine.

[CIT0008] Bitenc, R. (2020) Reconsidering dementia narratives: Empathy, identity and care. Routledge.

[CIT0009] Bitenc, R. (2012) Representations of dementia in narrative fiction. In Cohen, E. (Ed.), Knowledge and pain. (pp. 305–329). Rodopi.

[CIT0010] Carson, J. & Lugea, J. (eds.) (2022). A little unsteadily into the light. New Island.

[CIT0011] Clarke, J. (2006). The case of the missing person: Alzheimer’s disease in mass print Magazines 1991–2001. Health Commun, 19(3), 269–276. doi:10.1207/s15327027hc1903_9 16719730

[CIT0012] Davies, T., Houston, A., Gordon, H., McLintock, M., Mitchell, W., Rook, G., & Shakespeare, T. (2021). Dementia enquirers: Pioneering approaches to dementia research in UK. Disability & Society, 37(1), 129–147. doi:10.1080/09687599.2021.1916887

[CIT0013] Dean, D. (2006) Madonnas of Leningrad. William Morrow.

[CIT0014] DEEP. (2013, December 2022). DEEP Guide: Writing dementia-friendly information. DEEP: The dementia engagement and empowerment project. https://www.dementiavoices.org.uk/wp-content/uploads/2013/11/DEEP-Guide-Writing-dementia-friendly-information.pdf.

[CIT0015] Eder, J., Jannidis, F. & Schneider, R. (2010). Characters in fictional worlds: Understanding imaginary beings in literature, film, and other media. De Gruyter. doi:10.1515/9783110232424.1.3

[CIT0016] Fernandez-Quintanilla, C. (2020). Textual and reader factors in narrative empathy: An empirical reader response study using focus groups. Language and Literature, 29(2), 124–146. doi:10.1177/0963947020927134

[CIT0017] Genova, L. (2007) Still Alice. Simon & Schuster.

[CIT0018] Hall G. M. (2008) Empirical research into the processing of free indirect discourse and the imperative of ecological validity. In S. Zyngier, M. Bortolussi, A. Chesnokova, & J. Auracher (Eds.) Directions in empirical literary studies in honour of Willie Van Peer. (pp. 21–34). John Benjamins.

[CIT0019] Harvey, S. (2010) The Wilderness. Vintage.

[CIT0020] Harvey, K., & Brookes, G. (2019). Looking through dementia: what do commercial stock images tell us about aging and cognitive decline? Qualitative Health Research, 29(7), 987–1003. doi:10.1177/1049732318814542 30522392

[CIT0021] Healey, E. (2015) Elizabeth is Missing. Penguin Books.

[CIT0022] Hepworth, S. (2017) The things we keep. St. Martin’s Griffin.

[CIT0023] Kitwood, T. (1997) Dementia reconsidered: The person comes first. Open University.

[CIT0024] LaPlante, A. (2012) Turn of mind. Vintage.

[CIT0025] Lugea, J. (2022). Dementia mind styles in contemporary narrative fiction. Language and Literature, 31(2), 168–195. doi:10.1177/09639470221090386

[CIT0026] Northern Ireland Statistics and Research Agency (2022). Census 2021—Country of birth. https://www.nisra.gov.uk/publications/census-2021-main-statistics-demography-tables-country-of-birth.

[CIT0027] Palmer. (2004) Fictional minds. University of Nebraska.

[CIT0028] Rill, E. (2015) An Absent Mind. Lake Union Publishing.

[CIT0029] Rimkeit, B.S. & Claridge, G. (2017) Peer reviewed: literary Alzheimer’s, a qualitative feasibility study of dementia-friendly book groups. New Zealand Library & Information Management Journal, 56(2), 14–22. doi:10.18438/eblip29417

[CIT0030] Shakespeare, T., Zeilig, H., & Mittler, P. (2019). Rights in mind: Thinking differently about dementia. Dementia, 18(3), 1075–1088. doi:10.1177/1471301217701506 28387137

[CIT0031] Smith, A. (2012) There but for the. Anchor books.

[CIT0032] Swinnen, A. (2013). Dementia in documentary film: Mum by Adelheid Roosen. Gerontologist, 53(1), 113–122. doi:10.1093/geront/gns061 22539659

[CIT0033] Swinnen, A., & de Medeiros, K. (2018). ‘Play’ and people living with dementia: A humanities based inquiry of Timeslips and the Alzheimer’s Poetry Project. Gerontologist, 58(2), 261–269. doi:10.1093/geront/gnw196 28329857

[CIT0034] Twigg, J., & Martin, W. (2014). The challenge of cultural gerontology. Gerontologist, 55(3), 353–359. doi:10.1093/geront/gnu061 24974388

[CIT0035] Wade, M. (2015) *Still Alice, and the advocacy for Alzheimer’s in fiction*. The Conversation. https://theconversation.com/still-alice-and-the-advocacy-for-alzheimers-in-fiction-36757.

[CIT0036] Zeilig, H. (2013). Dementia as a cultural metaphor. Gerontologist, 54(2), 258–267. doi:10.1093/geront/gns203 23408265

