# Supplementary material for: “Sometimers, Alzheimer’s? I love that! That’s definitely me”: Readers’ Responses to Fictional Dementia Narratives
Source: Gerontologist. 2023 May 12;63(10):1610–8. doi: 10.1093/geront/gnad055 (PMC10724039; doi:10.1093/geront/gnad055)
Supplement: gnad055_suppl_Supplementary_Material [file gnad055_suppl_supplementary_material.docx]

**Online Supplementary Material**

**Supplement 1:** Coding of the group discussions

The group discussions were transcribed by professional transcribers and anonymized by the research team. The transcripts and recordings were uploaded into a shared project in *Atlas.ti*. First, authors 1 and 2 engaged in independent inductive coding of the transcripts of session 1 only. Next we merged the datasets and the whole team met at a face-to-face meeting to discuss the emergent codes. At this point we established a number of nascent code groups from both disciplinary perspectives (gerontology and linguistics). These were – codes pertaining to age and gender; memory and reminiscing; humor, lived experience of dementia and understanding of dementia. Linguistic codes referred to characterization; reader engagement with the extract and reading more generally; stylistic features and how the readers attributed characteristics to themselves, and to the character with dementia. Authors 1 and 2 then returned to independently coding sessions 2 to 6. We used these agreed code groups as a guide but continued to generate new inductive codes if the data presented new evidence. Next we had another all team meeting to discuss codes, offer explanations for other team members and to make some decisions about merging or cleaning the dataset. This was done in May 2022. By June we had the final master bundle which contains 189 codes, 13 memos and 102 documents (see supplementary material: Code List with Definitions). The references to codes take the following format: (Andy on Saul Reimer D8:149 – 64). Andy is a pseudonym for one of the readers with dementia. Saul Reimer is the character with dementia to which he refers in the quotation. D8 is the number of the document given to transcript 1a, Group D by Atlas.ti. 1a refers to the first extract discussed in week 1’s reading group session. Group D is readers with dementia. Group C were carers, Group B were the general public and Group A were student social workers. So, transcript 2b, Group B would refer to the second extract listened to by readers from the general public in week 2’s reading group session.

The supplementary material: Code List with Definitions shares a list of all codes generated by coders over our year-long independent coding of group discussions of all 12 extracts across all four reading groups.

**Explanation of terms and description of Table 2: Readers’ demographic characteristics**

Group A (Student Social Workers) were, by definition, all in full-time university study. Most were aged around 20 with two mature students in 39-49 age category. Three already had an undergraduate degree in another subject, which is usual for social work students.

Group B (General Public) were more diverse in age but more homogenous in terms of socio-economic status. All had A-level or above (pre-university qualifications) and three had postgraduate degrees. Occupations included psychotherapist, civil servant, shop owner and publishing.

Group C (Carers) were all over 40 but under 80. All had at least a high school diploma (GCSE or O-level), though only one had a university degree. Occupations included hospitality, engineering, human resources and nursing.

Group D (People with Dementia) were the most homogenous in terms of age (50-79) but the most diverse in terms of education. Three had no qualifications (i.e. no high school diploma) and one participant had a degree. Occupations included building, driving and disability support worker.

**Supplementary Table 2:** Code List with Definitions

| **Code** | **Comment** | **Code Group 1** | **Code Group 2** |
| --- | --- | --- | --- |
| AGE/GEN - # sandwich generation recognised | Statement explicitly pointing out how more than two generations may need care from middle generation (carers with children/grandchildren to care for). | Age and gender |  |
| AGE/GEN - #age as evidence of dementia | Reader mentions a person or character's age as a reason to assume that they have dementia. | Age and gender |  |
| AGE/GEN - #life course not just old person | Referring to some other or earlier stage of the lifecourse, not just old age. | Age and gender |  |
| AGE/GEN - #ref to own age or life stage | Some comment 'at my age' or 'now that I am retired' | Age and gender |  |
| AGE/GEN - hegemonic masculinity of character | Reader identifies how unreconstructed gender norms may be influencing male character or relative's response to dementia diagnosis. | Age and gender |  |
| AGE/GEN - memory/reminiscing | Participants comment on reminiscing (long-term memory) | Age and gender | Memory |
| AGE/GEN - ref to physicality man w/ dementia | Some reference made to the physicality of the man with dementia | Age and gender |  |
| AGE/GEN - ref to physicality woman w/ dementia | Some reference made to the physicality of the woman with dementia | Age and gender |  |
| AGE/GEN - time | Reader refers to either the passage of time or how time is used by the author. Any reference to time at all.    17/06/2022 09:02:30, merged with Participant refers to the passage of time in characters' lives Refers to the passage of time |  |  |
| AGEISM | this code captures  1) comments that relate to stereotypical attitudes around AGE and aren't covered by the other codes, and (analyst's interpretation) 2) whenever participants point out something as ageist  17/06/2022 09:10:40, merged with AGE/GEN - beliefs about old age_helpful  17/06/2022 09:10:40, merged with AGE/GEN - beliefs about old age_unhelpful | Age and gender |  |
| ATT factors - situational | "Participants attribute situational forces to characters’ actions and circumstances; i.e. they provide contextual explanations for characters’ behaviour..." (Fernandez-Quintanilla 2018: 207) | Attributing |  |
| ATT feeling to Carer | Feelings/emotions are attributed to CARERS only. |  |  |
| ATT Feeling to Character w Dementia | Participants attribute feelings to the character with dementia, whether made explicit in the extract, or imagined. | Attributing |  |
| ATT Feeling to other characters | Participants attribute feelings to the characters without dementia. THIS EXCLUDES CARERS! These feelings may be made explicit in the extract, or imagined. | Attributing |  |
| ATT Goals/Needs | Participants attribute goals and needs attributed to characters  (Fernandez-Quintanilla 2018: 208) | Attributing |  |
| ATT speech/thought affiliation | Participants verbally articulate characters’ speech/thought in direct form (Fernandez-Quintanilla 2018: 208).  e.g. "...it's almost as if he's proven, you know, 'I'm a tough guy, I can handle this'..." | Attributing |  |
| CHAR - schema refreshing | When participant notes that the character with dementia challenges preconceptions in some way i.e. they break the mould.  See Culpeper (2001) Language and Characterisation: People in Plays and Other Texts. | Characterisation |  |
| CHAR - schema reinforcing | When the character with dementia reinforces a participants preconceived ideas about dementia.    See Culpeper (2001) Language and Characterisation: People in Plays and Other Texts. | Characterisation |  |
| CHAR ATT - agency | The reader attributes the character withe dementia as having agency e.g. over their thoughts, actions, behaviours | Characterisation |  |
| CHAR ATT - childlike | When participants attribute a characteristic to the character with dementia - childlike | Characterisation |  |
| CHAR ATT - denial/failure to recognise own symptoms | Reference to pwd not being able to recognise own symptoms, mostly in groups C and D. |  |  |
| CHAR ATT - excluded | When participants notice that the character with dementia is excluded from interactions. | Characterisation |  |
| CHAR ATT - good long term memory | When participants observe the character with dementia has a good long-term memory. | Memory | Characterisation |
| CHAR ATT - honest/unfiltered |  |  |  |
| CHAR ATT - memory wins | 16/06/2022 10:29:44, merged with STYLE - memory wins Moments of recognition | Memory |  |
| CHAR ATT - paranoia/distrust |  | Characterisation |  |
| CHAR ATT - passive | When participants attribute the character with dementia with passivity. | Characterisation |  |
| CHAR ATT - perceptive | Reader notes that the character with dementia is 'perceptive' in some way.  e.g. to emotions or to his/environment | Characterisation |  |
| CHAR ATT - personhood | When participants observe that the character with dementia is more than their illness i.e. observe their abilities, skills, traits etc, regardless of dementia. | Characterisation |  |
| CHAR ATT - pride |  | Characterisation |  |
| CHAR ATT - rambling | When participants comment on the character with dementia’s incoherence or rambling talk. | Memory | Characterisation |
| CHAR ATT - resistant |  |  |  |
| CHAR ATT - self-deprecating |  | Characterisation |  |
| CHAR ATT - short term memory loss |  | Memory |  |
| CHAR ATT - strong | When participants attribute the character with dementia with strength. | Characterisation |  |
| CHAR ATT _ short-term memory loss |  |  |  |
| CHAR ATT loss of social status | This code is for when a reader identifies the impact of dementia on the character's social status. As we develop higher level codes this could be used of evidence of how the extract is helping readers to think through what it might be like to have dementia - and therefore - educating them. |  |  |
| CHAR-READ - character-oriented PT | See Fernandez-Quintanilla (2018: 204):"verbal displays of perspective taking, and distinguished whether participants’perspective taking is self-oriented (PT Self-oriented) or character-oriented (PTCharacter-oriented) because this distinction is highly relevant in my definition of empathy (see 2.2.2). [...]an example of character-oriented perspective taking would be “I put myself in the protagonist’s situation” | Reader-Character Engagement |  |
| CHAR-READ - Feeling with/for character | Reader makes specific mention of feeling with/for a character | Reader-Character Engagement |  |
| CHAR-READ - interaction | Participant reports a strong interaction with the character - this might be later subsumed with RESPONSE_other | Reader-Character Engagement |  |
| CHAR-READ - self-oriented PT | See Fernandez-Quintanilla (2018: 203)  "An example of self-oriented perspective taking would be “since I’m not a mother my head is unable to conceive what I would feel if I lost a child” | Reader-Character Engagement |  |
| COMP - of extracts | Participant compares extract with another extract or text.  17/06/2022 09:06:08, merged with READ - cross-ref with other extract I created this category, but then realised it could be subsumed with COMP - of extracts | Comparison |  |
| COMP w/ real pers - character w/ dementia | When the participant compares the character with a 'real' person, living, imagined or archetypal.05/08/2021, 16:36, merged with# compare char with real life experience of dementiaIn this code a reader compares the character's experience with their own experience of dementia in real life, usually, but not always a family member which is why it is a separate code to fam dem exp. Evaluating the situation with real life experience. | Comparison |  |
| COMP w/ real pers - other character | When participant compares character (not w/ dementia, nor carer) with a person in real life. | Comparison |  |
| COMP w/ real person - caring character | When participant compares the caring character with a person in real life (not themself - that is a diff category). | Comparison |  |
| COMP w/ self - caring character | When participant compares the caring character with themself. | Comparison |  |
| COMP w/ self - character w/ dementia | When the participant compares the character with dementia (or an aspect of them) to themself. | Comparison |  |
| EMOTION talk | Participants talk about emotion. What kinds of knowledge and assumptions do they bring? (Top-down processing) |  |  |
| EMPATHY - explicit | Participant makes explicit mention of empathetic response to character. | Reader-Character Engagement |  |
| EMPATHY - feels what text describes |  |  |  |
| EMPATHY - similarity of experience |  |  |  |
| EMPATHY - understanding |  |  |  |
| EMPATHY - what it's like |  |  |  |
| Empathy, iconicity, simulation? |  |  |  |
| EVAL - of character: mixed |  |  |  |
| EVAL - of character: neg | A lot of participants are angry with the actions of different characters, very useful for gauging perspective on dementia experience. | Evaluation |  |
| EVAL - of character: pos | 25/02/2022, 13:52, merged with EVAL of Character POS. This is an additional code for when the participants ascribe positive value to actions of character. 16/06/2022 12:43:35, merged withEVAL - of character: pos (2)16/06/2022 12:43:57, merged withCHAR-READ - liking16/06/2022 12:48:50, merged withCHAR ATT – humour. When participants observe that the character with dementia is humorous. | Evaluation |  |
| EVAL - of text: mixed | Participant gives a mixed evaluation of the extract, for whatever reason. | Evaluation |  |
| EVAL - of text: neg | Participant gives a negative evaluation of the extract. | Evaluation |  |
| EVAL - of text: pos | Participant gives a positive evaluation of the extract. | Evaluation |  |
| EVAL of Character POS | This is an additional code for when the participants ascribe positive value to actions of character. |  |  |
| EVAL – of care home: neg | Negative evaluation of care homes, either by character in extract or by participant in discussion. If needed, we can create sub-codes to accommodate the distinction — if meaningful later on. |  |  |
| EVAL – of carers: neg |  |  |  |
|  |  |  |  |
| HUM - appealing in characters | Readers identify how the humour of the character appeals to them. | Humour |  |
| HUM - function | Where participant describes how humour is used for a specific purpose in interaction -- in general, not just in the text. | Humour |  |
| HUM - function _ defence mechanism | Reader identifies how humour is used as defence against horror of dementia either in real life or extract. Or uses it themselves. | Humour |  |
| HUM - of participant | Evidence of humour or wit of reader.17/06/2022 09:11:34, merged withAGE/GEN - #self-mocking ageismReader uses humour as a defence mechanism to deflect ageism. | Humour |  |
| LIV EXP - Carers - feeling | This code refers to when a carer seems to make a plea to remember or acknowledge how hard it is for them.  LIVED EXPERIENCE - CARERS | Lived Experience |  |
| LIV EXP - Clash with experience | When the participant reports that an aspect of the text clashes with their experience. | Lived Experience |  |
| LIV EXP - fam dem exp | Straightforward code where reader mentions experience of caring for or visiting family member who has/had dementia. Not applicable to the carers group. | Lived Experience |  |
| LIV EXP - family conflict | Reference to disagreement amongst family members around dementia diagnosis/care etc.  FAMILY AS AN OVERARCHING CATEGORY? | Lived Experience |  |
| LIV EXP - family/carer perspective | Focused example or insight into dementia from a carer's perspective.   COMPARE SELF WITH CHARACTER IS OVERARCHING CATEGORY - WITHIN THAT, GIVE SUB-CATEGORIES FOR DIFFERENT PERMUTATIONS E.G. CARER COMPARES SELF TO CARING CHARACTER, CARER COMPARES SELF TO SHOP ASSISTANT ETC  06/08/2021, 16:27, merged with LIV EXP - Caring | Lived Experience |  |
| LIV EXP - Indirect Dementia | Revise/merge this category with other kinds of experience... | Lived Experience |  |
| LIV EXP - living with dementia | Reader who has a dementia diagnosis and mentions it in the group/preamble. Interesting to look at how people go about describing themselves/their illness. As I coded included specific references to living with dementia as there are important insights. Also, wide variety of forms of dementia are represented in our sample which is important.  LIVED EXPERIENCE - INC THERE  06/08/2021, 16:27, merged with LIV EXP - Direct Dementia | Lived Experience |  |
| LIV EXP - loss of some loved activity due to illness | Where reader refers to the loss of some activity they used to love doing but now cannot because of dementia (Groups C and D especially). | Lived Experience |  |
| LIV EXP - Other | Participant draws on any life experience -- e.g. "when I was in hospital compared to the characters in the care home". | Lived Experience |  |
| Loss of memory physically expressed through book |  |  |  |
| PART - expression of mutual support |  | Participant Interaction |  |
| PART DETAIL | When a participant tells us information about themselves which might help interpret their responses... some of these might need moved to Lived Experience | Participant Detail |  |
| PART DETAIL - personal interest in dementia for health | Participant Detail: Where a reader refers to their own possible future health and specifically likelihood of developing dementia. | Participant Detail |  |
| PART DETAIL - Prof interest (writer/book-seller/publisher) | PARTICIPANT DETAIL: Expresses some professional interest in reading. Mostly from GROUP B. | Participant Detail |  |
| PART DETAIL - refers to self as carer | PARTICIPANT DETAIL: Self-identifies as a carer for a pwd IRL | Participant Detail |  |
| Participant agreement | When participant express agreement with prior comments.  16/06/2022 10:58:48, merged with #inter-reader validation of real life experience One reader explicitly points out how another's perspective is correct. Strong agreement or offering of evidence to support claim of another reader. | Participant Interaction |  |
| Participant directly quotes from the extract |  |  |  |
| Participant disagreement | When a participant explicitly disagrees with another participant's response.  16/06/2022 10:54:48, merged with #Participant disagreement |  |  |
| READ - wants to read source book or not | Participant refers to the whole book, may be enquiring about reading the whole thing. | Reading |  |
| READ - difficulty with | Reader refers to struggling with reading, for any reason. INCLUDE IN OVERARCHING CATEGORY ABOUT READING INCLUDING MISUNDERSTANDING AND RELIVED EXPERIENCE AND FORGETTING THAT FICTION IS FICTION. | Reading |  |
| READ - gen interest in | Reader refers to general interest in reading for its own sake, or wanting to start reading more, or expand literary interest. | Reading | Participant Detail |
| READ - misunderstanding | 06/08/2021, 15:51, merged with # misunderstood some aspect of the extract When a reader misunderstands something (e.g. Jewish Canadian versus Jewish Comedian). | Reading |  |
| READ - motivations for |  | Reading |  |
| READ - relive experience | Explicit statement mentioning how reading an extract takes them back to a particular period of time or stage of living with dementia. | Reading |  |
| RESPONSE - comfort |  |  |  |
| RESPONSE - confused |  |  |  |
| RESPONSE - connects w/ experience |  |  |  |
| RESPONSE - not sorry |  |  |  |
| RESPONSE - other |  |  |  |
| RESPONSE - questions fictional account | Reader openly questions whether the fictional account of living with dementia is valid according to real life experience, I included discussions with facilitators to demonstrate how much we welcomed critique from the readers. |  |  |
| RESPONSE_aesthetic | The reader reports that the text evoked some kind of response in them for aesthetic reasons   This seems to be important to distinguish from a response that is about emotion, or relating to an aspect of the plot/character.   It might later be combined with code EVALUATION of text: positive. | Response |  |
| RESPONSE_emotional | 05/08/2021, 15:39, merged with pPart experience of sentiment 05/08/2021, 16:06, merged with #emotion triggered by extract/character. | Response |  |
| RESPONSE_imagining or hypothesizing | Where the reader imagines or hypothesizes event(s) or situations around the extract (e.g. before, in the future) | Response |  |
| RESPONSE_moral | The reader expresses a moral response to the character and/or their behaviour | Response |  |
| RESPONSE_relate | Participant reports identifying or relating with the character with dementia | Response |  |
| STYLE - body parts | Participant refers to textual reference on body parts |  |  |
| STYLE - catch now, think later |  |  |  |
| STYLE - dramatic irony | Participant notices where the readers know something a character does not. | Stylistic Features |  |
| STYLE - metaphors | Participant comments on metaphors in the extract. | Stylistic Features |  |
| STYLE - Narrative stance | The participant refers to the way the character with dementia narrates story. Participants comment on the narrative or narration or point of view in lay people's terms.  e.g "putting us into Maud's shoes" | Stylistic Features |  |
| STYLE - physical sensations | The participant makes reference to the fact that a character's physical sensations are heightened   (e.g. 'my stomach closes in on itself') | Stylistic Features |  |
| STYLE - pragmatic difficulties | Participant notices the text representing communicative difficulties. | Stylistic Features |  |
| STYLE - Represented Discourse | The participant makes reference to the fact that a character directly reports the speech or writing they experience.  This would tally with stylistic analysis. | Stylistic Features |  |
| STYLE - sensory descriptions | 06/08/2021, 16:04, merged with # sensory description of dementia Reference to light, sound or some other sensory issue with the experience of living with dementia, either by character or example from real life. | Stylistic Features |  |
| STYLE - temporal disruption | when a participant notices that the character or text has an unusual flow of time / perception of the passage of time | Stylistic Features |  |
| STYLE - underlexicalisation | 05/08/2021, 16:26, merged with # under-lexicalisation Reference to loss of specific or accurate language as a symptom of dementia. | Stylistic Features |  |
| UND - cog v. intelligence | This code refers to readers expressing of confusion around how cognitive impairment might manifest - how a person with dementia can still be intelligent. Displays confusion around how we try to disassociate the person with dementia from their symptoms, or how we accept that they become their illness, and nothing more.  SUB-CODE to PERSONHOOD | Understanding of Dementia |  |
| UND - insight into character with dementia's/pwd's perspective | Focused or elaborate account of the person with dementia's experience either character or IRL. Slightly confused with other similar code, need to clarify in session. | Understanding of Dementia |  |
| UND - Learning/awareness | 05/08/2021, 15:17, merged with #learned from extract Explicit statement saying that the reader learned from the extract  16/06/2022 11:00:35, merged with #identifying how literary language adds to understanding of dementia | Understanding of Dementia |  |
| UND - metaphor used by participant | The reader shares a phrase or metaphor from their experience, possibly like one that appears in the extract. Or, they may point out a metaphor in the text. |  |  |
| UND - need for awareness | Comments on the lack or need for awareness around dementia.06/08/2021, 16:21, merged with# lack of awareness of dementiaLack of awareness of how dementia manifests and how people with dementia should be treated. General public and/or individual characters or people IRL. AWARENESS - OVERARCHING CODE, INC LACK OF PUBLIC AW, OR INVISIBLE DISABILITIES, LACK OF TRAINING, READER DEMONSTRACTING/EXP STATING LACK OF AWARENESS06/08/2021, 16:28, merged with#ignorance about dementiaReader makes some reference to lack of knowledge about dementia when a character displays a trait or does something s/he finds surprising. This raises interesting questions for us about whether the author's depiction is 'accurate' in a literal sense. Most evident in the carers group, who question some of the internal monologues and alleged actions of characters with dementia. Groups A and B talk about their own ignorance of dementia and GROUPS C and D about the ignorance of the general public, see my memo. We are definitely onto something important with this study design. Potentially important for follow on study too. | Understanding of Dementia |  |
| UND - prevalence of dementia/indirect consequences | Reference to the fact that 'no one is untouched by dementia' - the idea that it is so prevalent that it affects us all. | Understanding of Dementia |  |
| UND - Strategies for dealing with Dementia | Participant describes strategies for dealing with dementia, which may or may not be inspired by the extract. | Understanding of Dementia |  |
| UND_ sceptic of real understanding |  | Understanding of Dementia |  |
